# Activation of AMP-Activated Protein Kinase **α** and Extracelluar Signal-Regulated Kinase Mediates CB-PIC-Induced Apoptosis in Hypoxic SW620 Colorectal Cancer Cells

**DOI:** 10.1155/2013/974313

**Published:** 2013-03-26

**Authors:** Sung-Yun Cho, Hyo-Jeong Lee, Hyo-Jung Lee, Deok-Beom Jung, Hyunseok Kim, Eun Jung Sohn, Bonglee Kim, Ji Hoon Jung, Byoung-Mog Kwon, Sung-Hoon Kim

**Affiliations:** ^1^College of Oriental Medicine, Kyung Hee University, 1 Hoegi-dong, Dongdaemun-gu, Seoul 130-701, Republic of Korea; ^2^Korea Research Institute of Bioscience and Biotechnology, University of Science and Technology, 52 Uendong, Yuseonggu, Daejon 305-806, Republic of Korea; ^3^Cancer Preventive Material Development Research Center, College of Oriental Medicine, Kyung Hee University, 1 Hoegi-dong, Dongdaemun-gu, Seoul 130-701, Republic of Korea

## Abstract

Here, antitumor mechanism of cinnamaldehyde derivative CB-PIC was elucidated in human SW620 colon cancer cells. CB-PIC significantly exerted cytotoxicity, increased sub-G1 accumulation, and cleaved PARP with apoptotic features, while it enhanced the phosphorylation of AMPK alpha and ACC as well as activated the ERK in hypoxic SW620 cells. Furthermore, CB-PIC suppressed the expression of HIF1 alpha, Akt, and mTOR and activated the AMPK phosphorylation in hypoxic SW620 cells. Conversely, silencing of AMPK**α** blocked PARP cleavage and ERK activation induced by CB-PIC, while ERK inhibitor PD 98059 attenuated the phosphorylation of AMPK**α** in hypoxic SW620 cells, implying cross-talk between ERK and AMPK**α**. Furthermore, cotreatment of CB-PIC and metformin enhanced the inhibition of HIF1**α** and Akt/mTOR and the activation of AMPK**α** and pACC in hypoxic SW620 cells. In addition, CB-PIC suppressed the growth of SW620 cells inoculated in BALB/c athymic nude mice, and immunohistochemistry revealed that CB-PIC treatment attenuated the expression of Ki-67, CD34, and CAIX and increased the expression of pAMPK**α** in CB-PIC-treated group. Interestingly, CP-PIC showed better antitumor activity in SW620 colon cancer cells under hypoxia than under normoxia, since it may be applied to chemoresistance. Overall, our findings suggest that activation of AMPK**α** and ERK mediates CB-PIC-induced apoptosis in hypoxic SW620 colon cancer cells.

## 1. Introduction

Hypoxia is a critical factor of solid tumors that renders tumor cells resistant to some chemotherapeutic agents [1]. Most of the cancer cells grow fast and suffer nutrition and oxygen deficiency under hypoxia [2]. Under hypoxic condition, the cells are regulated by HIF-1*α* and other related signaling including AMP-activated protein kinase (AMPK) pathway. AMPK consists of *α* catalytic subunit and regulatory *β* and *γ* subunits, which act for cellular adaptation to ATP-consuming stimuli such as glucose deprivation [3]. When cells need to overcome metabolic stress, the ratio of AMP to ATP is markedly increased by AMPK [4]. Thus, activation of AMPK suppresses metabolic functions that consume or generate ATP [5, 6], and decreased activation of AMPK significantly increases the risk of cancer in several animal models [7–9]. Also, the activation of AMPK suppresses survival signals such as phosphoinositide-3-kinase (PI3K)/AKT signaling and regulates mTOR pathway [10]. Recently, metformin as an old antidiabetic drug emerges as anticancer agent via AMPK inhibition [11].

Mitogen-activated protein kinase (MAPK) pathways including c-Jun N-terminal kinase (JNK), extracellular signal-regulated kinase (ERK), and p38 are involved in the proliferation, differentiation, growth, and apoptosis of cells [12–14]. Among MAPK proteins, ERK is known to usually regulate cell proliferation or sometimes induces apoptosis [12]. Although cinnamaldehyde, a major compound of Cinnamon, was known to have antioxidant [15] and antitumor activities via regulation of peroxisome proliferator-activated receptor-*γ* (PPAR*γ*) and AMPK pathways [16, 17], its underlying mechanism still remains unclear. Thus, in the present study, the antitumor mechanism of cinnamaldehyde derivative CB-PIC was investigated in SW620 colorectal adenocarcinoma cells under hypoxia in association of AMPK and ERK signaling *in vitro* and *in vivo*. 

## 2. Materials and Method

### 2.1. Chemicals and Reagents

**Figure 1:**
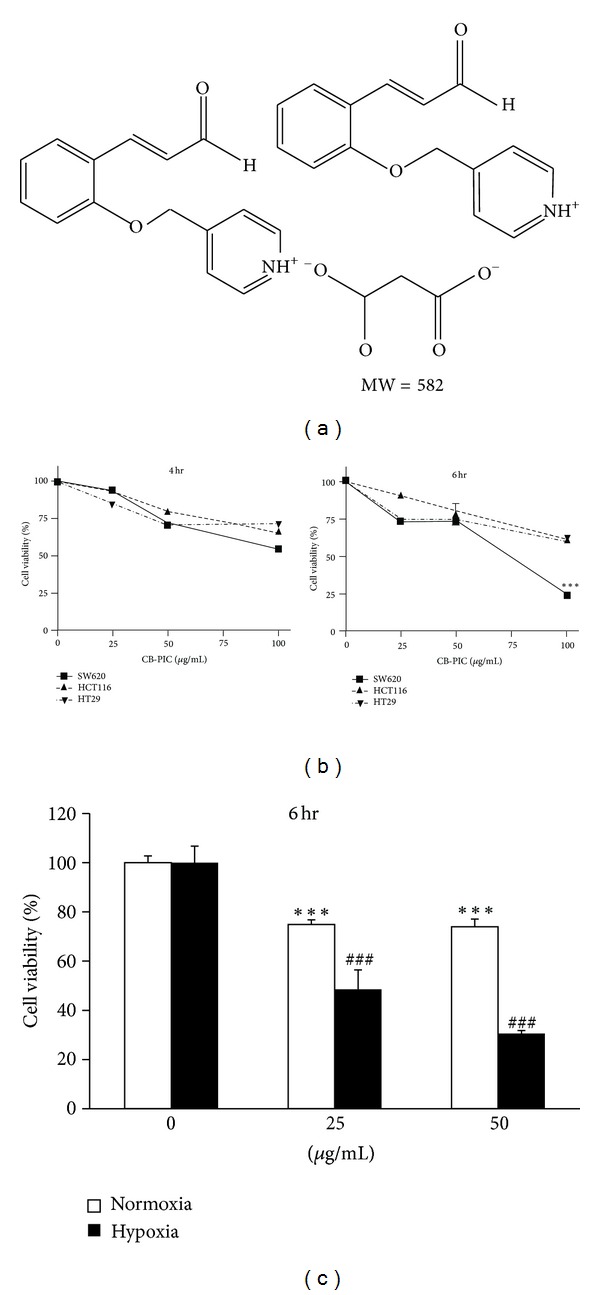
(a) Chemical structure of CB-PIC. Molecular weight = 582. (b) SW620, HC116, and HT29 cells were treated with various concentrations of CB-PIC (0, 25, 50, or 100 *μ*g/mL) for 4 and 6 hr. Cell viability was analyzed by MTT assay. (c) Cells were exposed to normoxia or hypoxia for 6 hours with CB-PIC (0, 25 or 50 *μ*g/mL). Data are presented as means ± SD. ***P < 0.001 compared with control and ^###^ *P* < 0.001 compared with hypoxia control.

(E)-4-((2-(3-oxopop-1-enyl)phenoxy)methyl)pyridinium malonic acid (CB-PIC; C_33_H_30_N_2_O_8_; MW = 582.59) was kindly given from Dr. Byung-Mog Kwon's lab, (Korea Research Institute of Bioscience and Biotechnology, Daejeon, Korea) (Figure 1(a)). SW620 human colorectal adenocarcinoma cells (American Type Culture Collection, Manassas, VA, USA) were maintained in RPMI 1640 supplemented with fetal bovine serum (FBS), liquid gentamicin reagent solution, penicillin and streptomycin, and trypsin EDTA (Gibco Carlsbad, CA, USA). Enhanced chemiluminescence (ECL) Western blotting detection reagents and Hyperfilm ECL were purchased from Amersham-Pharmacia (Amersham-Pharmacia Korea, Seoul, Korea). Anti-rabbit IgG heavy and light chain-specific (rabbit, mouse) peroxidase conjugates and antibody against HIF-1*α* were purchased from Gentex (Irvine, CA, USA). Antibodies against, pAKT, AKT, ERK, pERK, pACC, p-mTOR, cleaved caspase-3, PARP, pAMPK*α*, and AMPK*α* were from Cell Signaling Technology (Denver, MA, USA). Antibodies against *β*-actin and metformin (Cas no. 1115-70-4) were purchased from Sigma Chemical Co. (St. Louis, MO, USA). The siRNA transfection reagent was from Polyplus-transfection (Illkirch, France). Control siRNA and AMPK*α* siRNA were purchased from Santa Cruz Biotechnology (Santa Cruz, CA, USA). 

### 2.2. Cell Culture under Hypoxia

SW620 cells were seeded in 100 mm Falcon plates at 1 × 10^6^ cells/plate in RPMI 1640 supplemented with 10% FBS and 1% penicillin/streptomycin. The cells were cultured at 37°C in a humidified atmosphere containing from 5% CO_2_ to 60–80% confluence and then used for Western blot analysis. For the hypoxic treatment, the cells were transferred under an anaerobic chamber (Forma Scientific, OH, USA) with a humidified atmosphere of 2% O_2_ and 5% CO_2_ balanced with N_2_ and exposed to various concentrations of CB-PIC for 2~24 hours.

### 2.3. Cytotoxicity Assay

Cytotoxicity of CB-PIC was evaluated by 3-(4,5-dimethylthiazol-2-yl)-2,5-diphenyl tetrazolium bromide (MTT) assay. Briefly, cells were seeded onto 96-well microplates at a density of 2 × 10^4^ cells per well and treated with various concentrations of CB-PIC (0, 20, 50, or 100 *μ*g/mL) for 4, 6, or 24 hours. After indicated incubation times, MTT (1 mg/mL) (Sigma Chemical Co., St. Louis, MO, USA) solution was added for 2 hours, and MTT lysis buffer (20% SDS and 50% dimethylformamide) was then added for overnight. Optical density (OD) was measured using a microplate reader (TECAN, Austria) at 570 nm. Cell viability was calculated as a percentage of viable cells in CB-PIC-treated group versus untreated control by following equation:

(1) cell  viability  (%)  =[O.D.(CB−PIC)−O.D.(Blank)][O.D.(Control)−O.D.(Blank)]×100.

### 2.4. Terminal Deoxynucleotidyl Transferase dUTP Nick End Labeling (TUNEL) Assay

DNA fragmentation was analyzed by using DeadEnd fluorometric TUNEL assay kit (Promega, Madison, WI, USA). SW620 cells  (1 × 10^6^ cells/mL) were treated with 40 *μ*g/mL of CB-PIC for 6 hours under hypoxia chamber. After incubation with CB-PIC, cells were plated onto poly-L-lysine-coated slide. The cells were fixed in 4% methanol-free formaldehyde solution (PBS, pH 7.4) for 30 min at 4°C. After fixation, most steps were carried out according to the manufacturer's protocol. Briefly, terminal deoxyribonucleotidyl transferase (TdT) enzyme buffer containing fluorescein-12-dUTP was added to the plates for 1 hour at 37°C in a dark humidity chamber. The slides were mounted with mounting medium containing 40,6-diamidino-2-phenylindole (DAPI) (Vector Laboratories, Burlingame, CA, USA) and visualized under an Olympus FV10C-W confocal microscope (Olympus, USA).

### 2.5. Cell Cycle Analysis

SW620 cells were treated with CB-PIC (4 *μ*g/mL) for 2, 4, 6, and 8 hours under hypoxia. The cells were fixed in 75% ethanol at −20°C and treated with RNase A (10 mg/mL) for 1 h at 37°C, stained with propidium iodide (PI) (50 *μ*g/mL) and analyzed for the DNA content by FACSCalibur (Becton-Dickinson, Franklin Lakes, NJ, USA) using CellQuest Software (BD Bio-sciences, San Jose, CA, USA). 

### 2.6. Caspase-3 Colorimetric Assay

Cells (1 × 10^6^ cells) treated with CB-PIC for 6 hours were measured enzyme activity of the caspase-3 class of protease in apoptotic cells by using Caspase-3 Colorimetric Assay Kit (R&D Systems, Minneapolis, MN, USA) according to manufacturer instructions.

### 2.7. Western Blotting

Cells (1 × 10^6^ cells) treated with CB-PIC, metformin, and PD98059 (ERK inhibitor) were lyzed by using lysis buffer (50 mM Tris-HCl, pH 7.4, 300 mM NaCl, 0.5% Triton X-100, 0.1% SDS, 5 mM EDTA, and protease inhibitor cocktail). The extracts were incubated on ice for 30 min, at 14,000 ×g for 30 min at 4°C, and the supernatants were collected for Western blotting. Protein concentrations were determined by Bradford assay (Bio-Rad), and equal amounts of proteins (30 *μ*g) were separated by electrophoresis sodium dodesyl sulfate polyacrylamide gel electrophoresis (SDS-PAGE) and transferred to PVDF membranes (Amersham Biosciences, Piscataway, NJ, USA). The membranes were blocked with 5% skim milk in Tris-buffered saline containing 0.1% Tween 20 for 2 hours at room temperature. The membranes were probed overnight at 4°C with mouse anti-human HIF-1*α* (1 : 1000 dilution; Genetex, Irvine, CA, USA) and mouse anti-human *β*-actin(1 : 1000; Sigma Aldrich, St. Louis, MO, USA), anti-human pAKT, AKT, ERK, pERK, pACC, pmTOR, pAMPK*α*, AMPK*α*, PARP, cleaved caspase-3, and BCl-2 (1 : 1000; Cell Signaling Technology, Danvers, MA, USA), followed by washing and incubation with HRP-conjugated secondary antibody (Abd Serotec, Raleigh, NC, USA). Immunoreactive bands were visualized using the ECL system (Amersham-Pharmacia, Seoul, Korea).

### 2.8. AMPK*α* Transfection Assay

SW620 cells were transfected with siRNA control or AMPK*α* by using Polyplus siRNA transfection reagent (Illkirch, France) according to manufacturer instructions and then treated with CB-PIC for 4 hr under hypoxia. In brief, siRNA (100 pmol) was mixed with transfection reagent in Opti-MEM serum-free media (Invitrogen) and incubated for 15 min at room temperature. The siRNA/transfection reagent mixture was added to the cells for 48 hr. Medium was changed before CB-PIC treatment under hypoxia.

### 2.9. Animals and CB-PIC Treatment

Six-week-old male BALB/c athymic nude mice (25 ± 3 g) were purchased from Chung-Ang Laboratory Animals (Seoul, Korea) and housed in animal facility at 22 ± 3°C and 60 ± 10% humidity with light-controlled (12 h, 07:00–19:00) environment. All materials including bedding and feed were sterilely cleaned by UV rays for 15 min before treated to the mice. The experiments were conducted in accordance with the guidelines approved by Institutional Animal Care and Use Committee, Kyung Hee University (KHUASP(SE)-11-005). CB-PIC was diluted in 4% Tween 20 normal saline. After 1 week adaptation, the animals were assigned to four groups (*n* = 6): normal control (vehicle), negative control (vehicle (saline) + SW620 inoculation), CB-PIC20 (20 mg/kg + SW620 inoculation), and CB-PIC50 (50 mg/kg + SW620 inoculation). CB-PIC solved in saline and Tween 20 was daily injected into the abdomen of mice for 20 days. 

### 2.10. SW 620 Xenograft Model

The animal study was conducted under guidelines approved by Institutional Animal Care and use Committee, Kyung Hee University [KHUASP(SE)-11-005] as previously described with minor modifications [18]. Briefly, 2 × 10^6^ of SW 620 cells were mixed with Matrigel (Becton Dickinson, 50% in 100 *μ*L) and injected subcutaneously into the right flank of 6-week-old male BALB/c athymic nude mice (Chung-Ang Laboratory Animals, Seoul, Korea)) for 3 groups (Control and CB-PIC groups). Three day after SW620 cell inoculation, 2% Tween-80/saline was daily injected into the mice of control group, while CB-PIC (20 and 50 mg/kg) dissolved in 2% Tween-20 was injected into the mice of CB-PIC groups. Tumor size was monitored twice a week with a caliper, and tumor volume was also calculated as described [18]. At the end of animal study, tumors were dissected, weighed, and photographed. A piece of each tumor was fixed in 10% phosphate-buffered formalin (PBS) for immunohistochemistry. 

### 2.11. Immunohistochemistry

For immunohistochemistry, paraffin sections (4 *μ*m) from tumors dissected were stained with hematoxylin and eosin. Immunohistochemical staining was performed in tumor sections using the indirect avidin biotin-enhanced horseradish-peroxidase method. Antigen retrieval was performed after dewaxing and dehydration of the tissue sections by microwave for 10 min in 10 mM citrate buffer. Tumor sections were cooled at room temperature, treated with 3% hydrogen peroxide in methanol for 10 min, and blocked with 6% horse serum for 30 min at room temperature in humidity chamber. Sections were then incubated with the primary antibody against to Ki-67 (diluted 1 : 200, Lab Vision Corporation, Fremont, CA, USA), CAIX (Novus Biologicals, Littleton, CO, USA), AMPK*α* (Cell Signaling Technology, Denver, CO, USA) and CD34 (diluted 1 : 50, Abcam, Boston, MA, USA) at 4°C overnight in humidity chamber. Sections were washed in PBS and incubated with secondary antibody (biotinylated goat anti-rabbit (1 : 150, Vector Laboratories, Burlingame, CA, USA) or biotinylated rabbit anti-rat IgG (1 : 150, Abcam, Boston, MA, USA) for 30 min in humidity chamber. After further washes, the antibodies were detected with the Vector ABC complex/horseradish peroxidase (HRP) kit (Vector Laboratories, Burlingame, CA, USA), and color developed with 3,3′-diaminobenzidine tetrahydrochloride. For semiquantitation, ten photomicrographs (200×) were taken with a CCD camera, avoiding gross necrotic areas. The positively stained cells/vessels within each photomicrograph were counted. The counting of total cancer cells was aided with the ImagePro+ image processing program. 

### 2.12. Data Analyses

Every experiment was repeated at least three times. Data were shown as means ± SE. Significant differences were evaluated using Student's *t*-test and a Tukey-Kramer multiple comparison posttest.

## 3. Results

### 3.1. CB-PIC Exerts Significant Cytotoxicity in Hypoxic SW620 Cancer Cells

The hypoxic cells are considered more aggressive and resistant to various therapies including radiation or chemotherapy [19, 20]. Therefore, the cytotoxic effects of CB-PIC were evaluated in human SW 620 colorectal cancer cells under hypoxia or normoxia. CB-PIC showed significant cytotoxicity in SW 620 cells better than in HT29 and HCT116 cells (Figure 1(b)). Interestingly, the proliferation of SW 620 cells was increased under hypoxia compared to normoxia. However, CB-PIC dramatically suppressed the viability of SW620 cells even under hypoxia (Figure 1(c)). 

### 3.2. CB-PIC Induces Apoptosis in Hypoxic SW620 Cancer Cells

**Figure 2:**
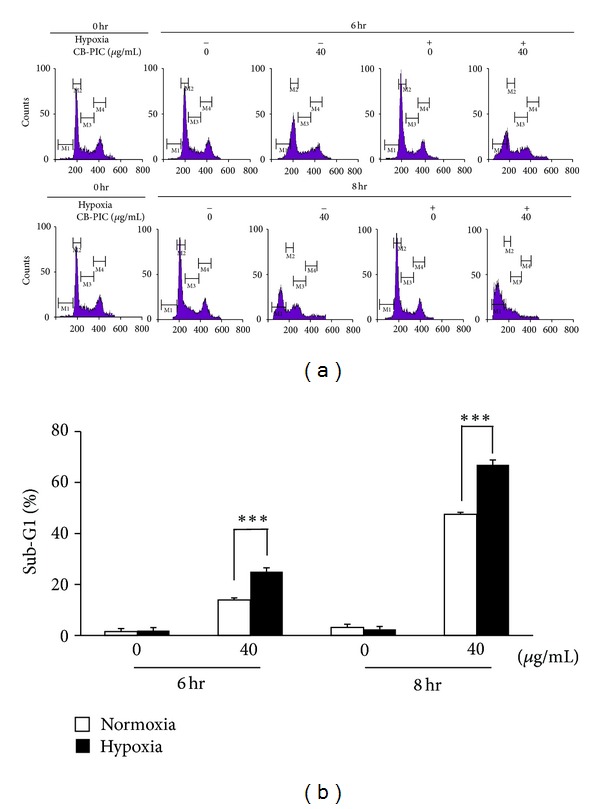
(a) Cells were treated with CB-PIC (0 or 40 *μ*g/mL) under normoxia and hypoxia both for 6 and 8 hr. Cell cycle distribution was analyzed by flow cytometry. (b) Bar graphs represent the percentage of the sub-G1 of apoptotic DNA fraction. Data are presented as means ± SD. ***P < 0.001 compared with normoxia CB-PIC-(40 *μ*g/mL) treated groups.

**Figure 3:**
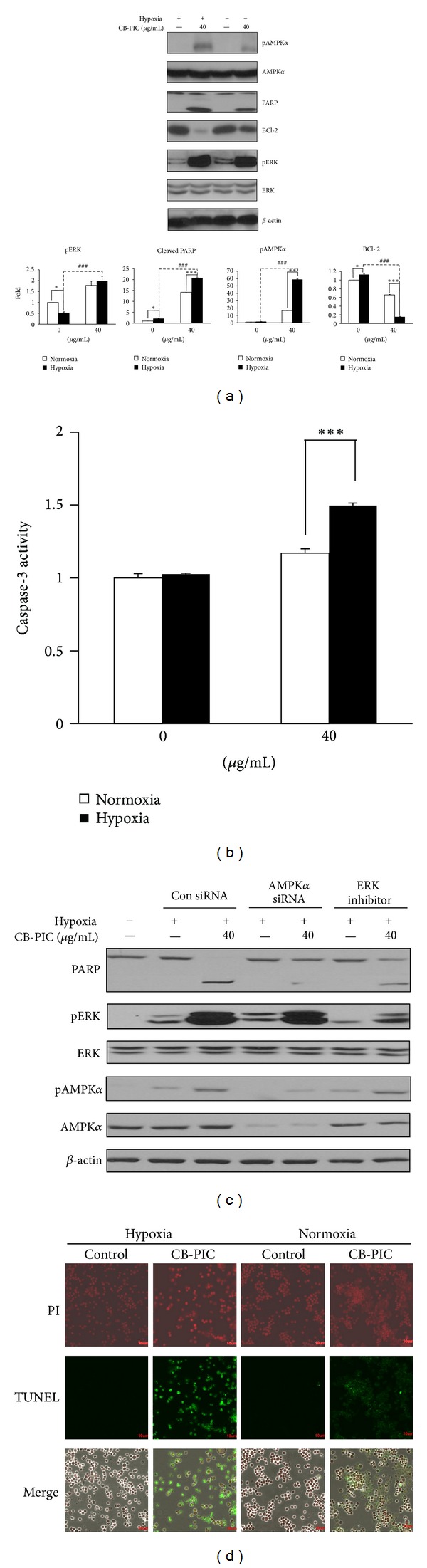
Cells were treated with or without CB-PIC (0 or 40 *μ*g/mL) under normoxic or hypoxic conditions for 6 hr. (a) Cell lysates were prepared and subjected to Western blotting to determine the expression of AMPK*α*, pAMPK*α*, PARP, BCl-2, pERK, ERK, and *β*-actin. Band density of pAMPK*α*, cleaved PARP, BCl-2, and pERK were quantified using Gel-pro analyzer (Media Cybernetics, Bethesda, MD, USA). Values are means ± SD, *n* = 3. *P < 0.05 and ***P < 0.001 compared with normoxia and hypoxia groups. (b) Cells (1 × 10^6^ cells) treated with CB-PIC for 6 hours were measured enzyme activity of the caspase-3 class of protease in apoptotic cells by using Caspase-3 Colorimetric Assay Kit. (c) Cells were transiently transfected with AMPK*α* siRNA or control siRNA in the presence or absence of CB-PIC (40 *μ*g/mL), and PD 98059 was also treated to SW 620 in the presence or absence of CB-PIC (40 *μ*g/mL) for 6 hours in hypoxia. Western blotting was performed to determine the expression of PARP, AMPK*α*, pAMPK*α*, pERK, ERK, and *β*-actin. (d) TUNEL staining was performed and visualized under fluorescence microscopy (×200).

To find out whether the cytotoxicity was due to apoptosis or necrosis, cell analysis was performed. CB-PIC significantly increased sub-G1 population in SW620 cells in a time-dependent manner in normoxia or hypoxia (Figures 2(a) and 2(b)). Consistently, CB-PIC significantly increased TUNEL positive cells (Figure 3(c)), cleaved PRAP, and attenuated the expression of Bcl-2 (Figure 3(a)) in SW620 cells under normoxia or hypoxia by Western blotting. In addition, the activity of the caspase-3 was increased by treatment of CB-PIC for 6 h in SW 620 cells (Figure 3(b)). 

### 3.3. CB-PIC Activated the Phosphorylation of AMPK*α* and ERK in Hypoxic SW620 Cancer Cells

**Figure 4:**
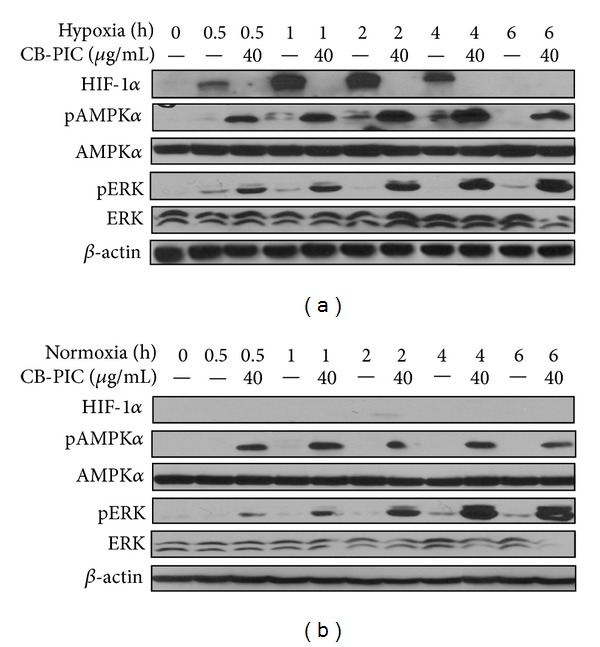
CB-PIC (0 or 40 *μ*g/mL) was treated under normoxic or hypoxic conditions for various times (0, 0.5, 1, 2, 4, or 6 hrs). Western blotting was performed to determine HIF-1*α*, AMPK*α*, pAMPK*α*, pERK, ERK, and *β*-actin expressions. (a) CB-PIC dramatically induces hypoxia-induced pAMPK*α* and pERK expressions and decreases hypoxia inducible factor 1*α* (HIF-1*α*) accumulation in SW620 cells under hypoxia as time-dependent manner. (b) CB-PIC induced pAMPK*α*, and pERK expression was increased.

It is well known that the decreased AMPK*α* activation is implicated in metabolic disorders with high cancer risk [21]. CB-PIC at 40 *μ*g/mL activated the phosphorylation of AMPK*α* and ERK in hypoxic SW620 cells (Figures 3(a) and 4(a)) and enhanced HIF1*α* accumulation up to 4 h in hypoxic SW620 cancer cells. In contrast, CB-PIC activated the phosphorylation of AMPK*α* and ERK both normoxia and hypoxia. Regarding the effect of ERK activation of cell cycle analysis and apoptosis, Tang and his colleagues suggested that low intensity DNA damage-induced ERK activation causes cell cycle arrest, while extensive DNA damage-induced ERK activation causes apoptosis [22]. CB-PIC induced PARP cleavage and ERK phosphorylation in SW620 cancer cells even after 6 h culture (Figure 3(a)). Similarly, CB-PIC at 40 *μ*g/mL activated the phosphorylation of AMPK and ERK in a time-dependent manner under hypoxia (Figure 4(a)) rather than under normoxia. However, HIF1*α* and pAMPK*α* tended to be downregulated after 4 h culture, while the phosphorylation of ERK was consistently increased by CB-PIC in hypoxic SW620 cells (Figure 4(a)). In contrast, pJNK and pp38 were not altered by CB-PIC under hypoxia and normoxia (data not shown). 

### 3.4. ERK Inhibitor PD98059 Attenuated Phosphorylation of AMPK*α* and Silencing of AMPK*α* Blocked Phosphorylation of ERK in Hypoxic SW620 Cancer Cells

**Figure 5:**
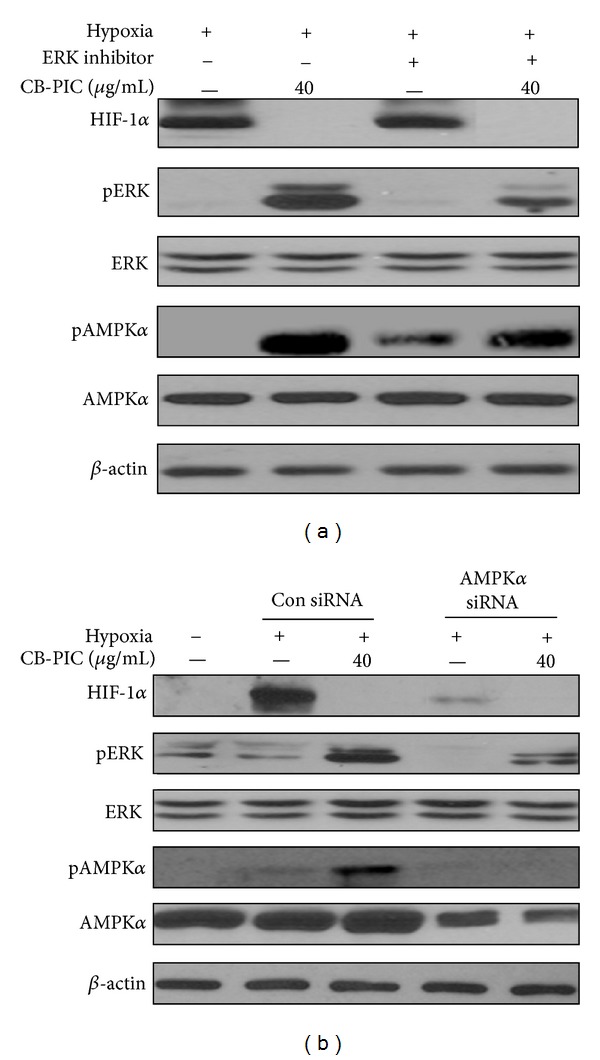
(a) SW620 cells were treated with CB-PIC (40 *μ*g/mL) and/or PD 98059(ERK inhibitor 5 *μ*M) for 2 hrs under hypoxia. Western blotting was performed to determine HIF-1*α*, AMPK*α*, pAMPK*α*, pERK, ERK, and *β*-actin expressions. (b) AMPK*α* siRNA decreases the activity of hypoxia-induced apoptosis in SW620 cells under hypoxia. Cells were transiently transfected with AMPK*α* siRNA or control siRNA in the presence or absence of CB-PIC (40 *μ*g/mL) under hypoxia. Western blotting was performed to determine the expression of HIF-1*α*, AMPK*α*, pAMPK*α*, pERK, ERK, and *β*-actin.

To elucidate the underlying mechanism between ERK and AMPK*α*, ERK inhibitor PD98059 and AMPK*α* siRNA transfection were used in hypoxic SW620 cells. ERK inhibitor PD98059 attenuated phosphorylation of AMPK*α* and ERK, while silencing of AMPK*α* blocked phosphorylation of ERK and AMPK*α* by CB-PIC in hypoxic SW620 cancer cells (Figures 5(a) and 5(b)).

### 3.5. CB-PIC Enhanced Phosphorylation of AMPK*α* and ERK Induced by Metformin in Hypoxic SW620 Cancer Cells

**Figure 6:**
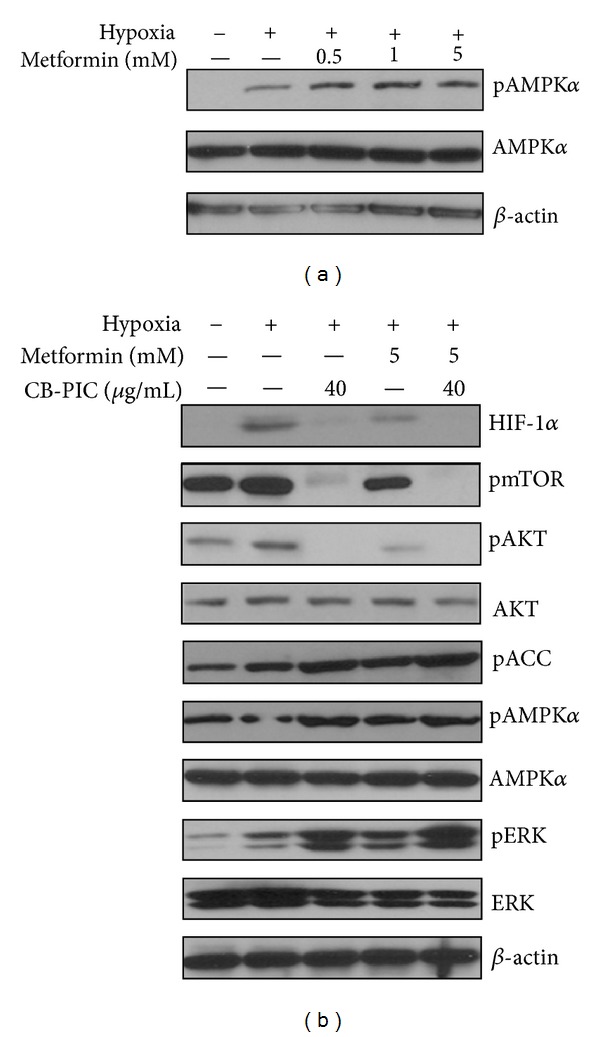
(a) Several concentrations of metformin (0, 0.5, 1, and 5 mM) were treated under hypoxia for 2 hr to determine the expression of AMPK*α*, pAMPK*α*, and *β*-actin. (b) SW 620 cells were treated with CB-PIC (40 *μ*g/mL) and/or metformin (5 mM) for 2 hr under hypoxia to investigate of effect of AMPK activator with CB-PIC (40 *μ*g/mL). Western blotting was performed to determine HIF-1*α*, AMPK*α*, pAMPK*α*, pERK, ERK, pmTOR, pAKT, AKT, pACC, and *β*-actin expressions.

As metformin is known as AMPK activator [23, 24] in SW620 cells under hypoxia, treatment with 5 mM metformin for 2 hours increased the phosphorylation of AMPK*α* (Figure 6(a)). In addition, cotreatment of CB-PIC and metformin enhanced phosphorylation of ACC, AMPK*α*, and pERK and also suppressed phosphorylation of mTOR and Akt and accumulation of HIF-1*α* in hypoxic SW620 cells (Figure 6(b)). 

### 3.6. CB-PIC Inhibited the Growth of SW620 Cancer Cells Inoculated in BALB/c Athymic Nude Mice

**Figure 7:**
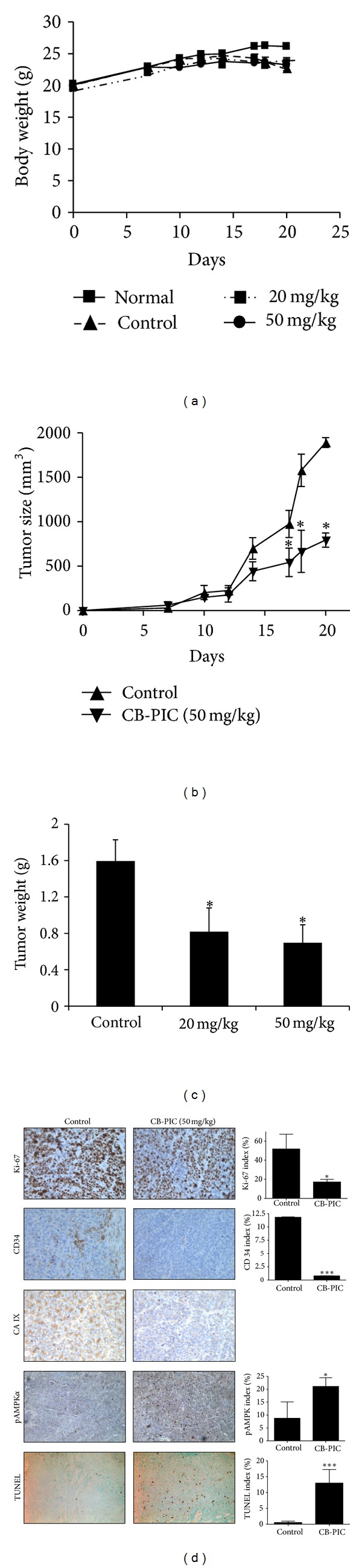
Colorectal cancer xenograft growth was suppressed by CB-PIC(20 and 50 mg/kg body weight) in female athymic nude mice. Starting three days after SW620 cell inoculation, CB-PIC (20 and 50 mg/kg body weight) was injected in abdomen with 4% Tween 20 as vehicle once daily. (a) Body weights of mice. (b) Tumor growth in a time course. (c) Final tumor weight at termination of experiment. (d) Representative examples of immunohistochemical staining for Ki-67, CD34, TUNEL, CA IX and pAMPK*α* in tumor sections. Graphs show the Ki67 index (proliferation), CD34 index (angiogenesis), TUNEL index (apoptosis), CA IX (hypoxia region), and pAMPK*α* index in tumor sections. Values are means ± SD, *n* = 6. *P < 0.05 and ***P < 0.001 compared with control mice.

CB-PIC suppressed the growth of SW620 cancer cells inoculated in BALB/c athymic nude mice at doses of 20 mg/kg and 50 mg/kg without affecting body weight (Figures 7(a), 7(b), and 7(c)). Consistently, immunohistochemistry revealed that CB-PIC treatment attenuated the expression of expression of Ki-67(proliferation), CD34 (blood vessel density), and carbonic anhydrase(CA)IX (hypoxic marker) and increased pAMPK*α* index in the tumor sections of CB-PIC-treated group (50 mg/kg) compared to untreated control (Figure 7(d)). 

## 4. Discussion

 Since clinically nearly 50~60% of solid tumors have more hypoxic regions compared to normal cells [25] and hypoxic cells are more aggressive and resistant to various cancer treatment [26, 27], it is crucial to regulate hypoxia signals. Thus, in the current study, molecular mechanism of CB-PIC was investigated in SW620 colon cancer cells under hypoxia. We found CB-PIC exerted significant cytotoxicity in SW620 cells better than HCT116 and HT29 colon cancer cells under hypoxia. We found the cytotoxicity of CB-PIC was caused by apoptosis, not necrosis, through the significant increase of sub-G1 accumulation, TUNEL positive cells, and PARP cleavage in hypoxic SW620 cells. 

AMP-activated protein kinase (AMPK), a cellular energy sensor conserved in eukaryotes, inhibits energy-consuming processes and activates energy-producing processes to restore the energy homeostasis inside the cell. Thus, AMPK activators, such as metformin and thiazolidinediones used for the treatment of type II diabetes, inhibit tumorigenesis through regulation of cell growth, cell proliferation, autophagy, stress responses, and cell polarity [5, 28]. Likewise CB-PIC enhanced the phosphorylation of AMP-activated protein kinase (AMPK) alpha and its substrate acetyl-CoA carboxylase (ACC) hypoxic SW620 colon cancer cells. ERK1/2 as one of MAPK proteins can be activated transiently or persistently by MEK1/2 and upstream MAP3Ks. In general, activation of ERK1/2 generally promotes cell survival, but under certain conditions, ERK1/2 can have proapoptotic functions [22, 29]. Also, some evidence suggests that ERK has function of proapoptotic characteristics under certain conditions, and other animal study supports that ERK can induce apoptosis [29, 30]. Activation of ERK can suppress the expression of phosphatidylinositol-3-kinase/ Akt survival pathway [31]. ERK and Akt reported to share some multimolecular complexes at least ERK1/2, Akt, ribosomal S6 kinase 1, and phosphoinositide-dependent kinase 1 [32]. In the present study, CB-PIC also activated ERK to induce apoptosis in hypoxic SW620 cells. 

Furthermore, CB-PIC suppressed the expression of hypoxia inducible factor 1 (HIF1) alpha as a hypoxia master regulator [33], Akt, and mammalian target of rapamycin (mTOR) similar to AMPK activator metformin in hypoxic SW620 cells, implying the important roles of mammalian target of rapamycin (mTOR) and AMPK*α* signaling in cancer metabolism [34, 35]. 

To find out the critical roles of AMPK*α* and ERK, ERK inhibitor PD 98059 and AMPK siRNA were used to evaluate their effects on CB-PIC-induced signaling in hypoxic SW620 cells. Silencing of AMPK*α* blocked PARP cleavage, and ERK activation induced by CB-PIC, while ERK inhibitor PD 98059 attenuated the phosphorylation of AMPK*α* in hypoxic SW620 cells, suggesting the possibility of cross-talk between ERK and AMPK*α*. Furthermore, cotreatment of CB-PIC and metformin enhanced inhibition of HIF1*α* and Akt/mTOR and activation of AMPK*α* and pACC in hypoxic SW620 cells.

 In addition, CB-PIC at 20 and 50 mg/kg suppressed the growth of SW620 cancer cells implanted in BALB/c athymic nude mice. Consistently, immunohistochemistry (IHC) showed that CB-PIC treatment attenuated the expression of decreased expression of Ki-67(proliferation), CD34 (blood vessel density), and carbonic anhydrase(CA)IX (hypoxic marker) and increased expression of pAMPK*α* index in CB-PIC-treated group compared to untreated control, indicating that CB-PIC exerts antitumor activity via inhibition of proliferation, angiogenesis, and hypoxia along with AMPK*α* activation and apoptosis induction in SW620 cancer cells. 

Given that HIF1*α*, a major target molecule in hypoxia is closely associated with multidrug resistance [36, 37], it is more significant that CB-PIC exerts antitumor activity in hypoxic SW620 cancer cells, since it can be applicable to cancer cells under hypoxic microenvironment with high risk of chemoresistance. Thus, it is also necessary for us to perform further study on the inhibitory effect of CB-PIC on MDR related proteins in resistant cancer cells *in vitro* and *in vivo* in the future. 

In summary, CB-PIC showed significant cytotoxicity against SW620 colon cancer cells and induced apoptosis by sub-G1 accumulation, the cleavage of PARP and caspase 3 and increased TUNEL positive cells in SW620 cancer cells. Interestingly, CB-PIC increased phosphorylation of ERK, AMPK*α*, and ACC, attenuated the expression of HIF1*α* and Akt/mTOR and also enhanced the antitumor activity of metformin by potentiating inhibition of HIF1*α* and Akt/mTOR and activation of AMPK*α* and pACC. Also, CB-PIC suppressed *in vivo* growth of SW620 cancer cells, and IHC showed decreased expression of Ki-67, CD34, and CAIX and increased expression of pAMPK*α*. Overall, our findings suggest that activation of AMPK*α* and ERK mediates CB-PIC-induced apoptosis in hypoxic SW620 colon cancer cells.
