# Corrigendum to “Activation of AMP-Activated Protein Kinase and Extracelluar Signal-Regulated Kinase Mediates CB-PIC-Induced Apoptosis in Hypoxic SW620 Colorectal Cancer Cells”

**DOI:** 10.1155/2020/6289392

**Published:** 2020-07-11

**Authors:** Sung-Yun Cho, Hyo-Jeong Lee, Hyo-Jung Lee, Deok-Beom Jung, Hyunseok Kim, Eun Jung Sohn, Bonglee Kim, Ji Hoon Jung, Byoung-Mog Kwon, Sung-Hoon Kim

**Affiliations:** ^1^College of Oriental Medicine, Kyung Hee University, 1 Hoegi-dong, Dongdaemun-gu, Seoul 130-701, Republic of Korea; ^2^Korea Research Institute of Bioscience and Biotechnology, University of Science and Technology, 52 Uendong, Yuseonggu, Daejon 305-806, Republic of Korea; ^3^Cancer Preventive Material Development Research Center, College of Oriental Medicine, Kyung Hee University, 1 Hoegi-dong, Dongdaemun-gu, Seoul 130-701, Republic of Korea

In the article titled “Activation of AMP-Activated Protein Kinase and Extracelluar Signal-Regulated Kinase Mediates CB-PIC-Induced Apoptosis in Hypoxic SW620 Colorectal Cancer Cells [1],” undeclared splicing was identified in the pERK bands, between lanes 5 and 6 in Figure 3(c) and HIF-1 alpha bands between lanes 3 and 4 in Figure 5(a).

The authors apologize for these errors which were due to mistakes during the cropping and naming of gels. As the original blots could not be obtained, the pERK, HIF-1, and actin gels were repeated, and new blots are being provided with the agreement of the editorial board. The new uncropped film data generated from the Gel doc system are also available in the supplementary files. These new results are consistent with the conclusions presented in the article. The updated figures with the new blots are shown as follows.

## Figures and Tables

**Figure 1:**
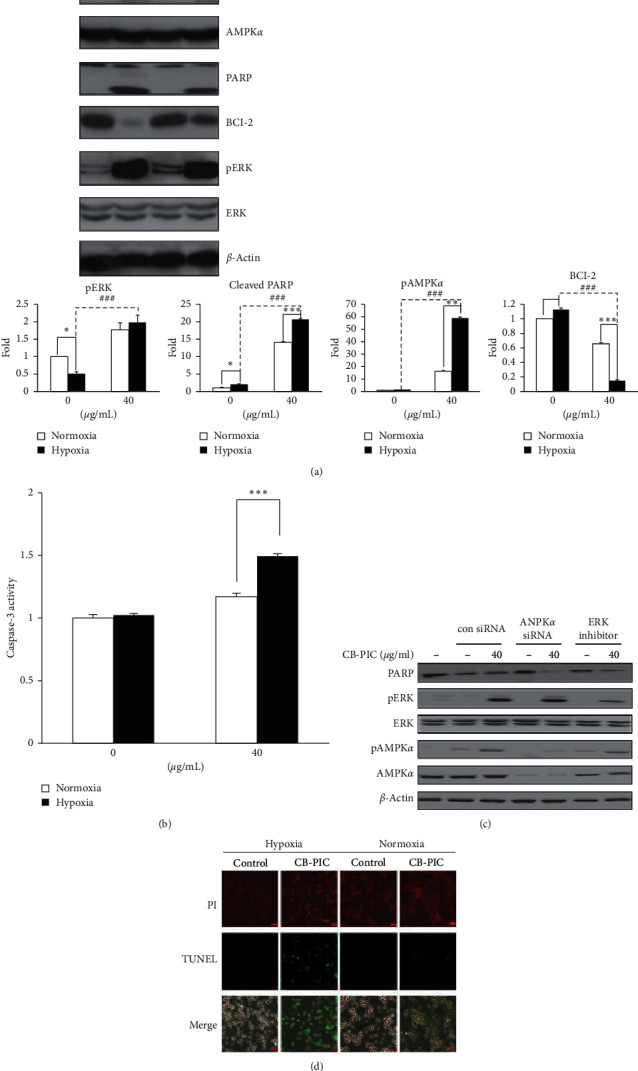
Cells were treated with or without CB-PIC (0 or 40 *μ*g/mL) under normoxic or hypoxic conditions for 6 h. (a) Cell lysates were prepared and subjected to western blotting to determine the expressions of AMPK*α*, pAMPK*α*, PARP, BCl-2, pERK, ERK, and *β*-actin. Band densities of pAMPK*α*, cleaved PARP, BCl-2, and pERK were quantified using Gel-pro analyzer (Media Cybernetics, Bethesda, MD, USA). Values are means ± SD, *n* = 3. ^*∗*^*P* < 0.05 and ^*∗∗∗*^*P* < 0.001 compared with normoxia and hypoxia groups. (b) Cells (1 × 106 cells) treated with CB-PIC for 6 hours were measured for enzyme activity of the caspase-3 class of protease in apoptotic cells by using the caspase-3 colorimetric assay kit. (c) Cells were transiently transfected with AMPK*α* siRNA or control siRNA in the presence or absence of CB-PIC (40 *μ*g/mL), and PD 98059 was also treated with SW 620 in the presence or absence of CB-PIC (40 *μ*g/mL) for 6 hours in hypoxia. Western blotting was performed to determine the expressions of PARP, AMPK*α*, pAMPK*α*, pERK, ERK, and *β*-actin. (d) TUNEL staining was performed and visualized under fluorescence microscopy (×200).

**Figure 2:**
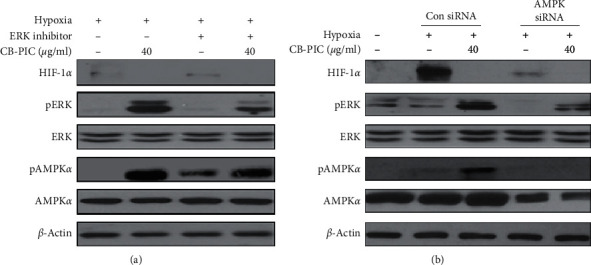
(a) SW620 cells were treated with CB-PIC (40 *μ*g/mL) and/or PD 98059 (ERK inhibitor 5 *μ*M) for 2 h under hypoxia. Western blotting was performed to determine HIF-1*α*, AMPK*α*, pAMPK*α*, pERK, ERK, and *β*-actin expressions. (b) AMPK*α* siRNA decreases the activity of hypoxia-induced apoptosis in SW620 cells under hypoxia. Cells were transiently transfected with AMPK*α* siRNA or control siRNA in the presence or absence of CB-PIC (40 *μ*g/mL) under hypoxia. Western blotting was performed to determine the expressions of HIF-1*α*, AMPK*α*, pAMPK*α*, pERK, ERK, and *β*-actin.
